# Appropriateness and clinical outcomes of short sustained low-efficiency dialysis: A national experience

**DOI:** 10.1515/med-2023-0868

**Published:** 2023-12-06

**Authors:** Alaa Rahhal, Mostafa Najim, Ahmed Mahfouz, Mhd Baraa Habib, Sara Seife Hassen, Isra’a Al-Shekh, Ashraf Omer Ahmed, Haneen Toba, Shahem Abbarh, Mawahib El Hassan, Sumaya Al Yafei, Amr Badr, Khaled Mohamed Mahmoud

**Affiliations:** Pharmacy Department, Hamad Medical Corporation, Doha, Qatar; Internal Medicine Department, Rochester Regional Health – Unity Hospital, New York, USA; Internal Medicine Department, Hamad Medical Corporation, Doha, Qatar; Critical Care Department, Hamad Medical Corporation, Doha, Qatar; Heart Failure Department, Hamad Medical Corporation, Doha, Qatar; Nephology Department, Hamad Medical Corporation, Doha, Qatar; Urology and Nephrology Center, Faculty of Medicine, Mansoura University, Mansoura, Egypt

**Keywords:** sustained low-efficiency dialysis, acute kidney injury, mortality, dialysis

## Abstract

Sustained low-efficiency dialysis (SLED) is usually performed over 6–12 h among hemodynamically unstable patients. Conduction of 4-h SLED may spare time and manpower during hospitalization. Therefore, we conducted a retrospective observational study to explore the appropriateness and clinical outcomes of 4-h SLED among critically ill patients admitted to our center from 1/06/2016 to 1/06/2020. Renal parameters including blood urea nitrogen, serum creatinine, sodium, phosphorus, potassium, and bicarbonate were determined on the day of dialysis before SLED and within 24 h after SLED, and clinical outcomes including, acute kidney injury (AKI) recovery, in-hospital mortality, 30-day mortality, 180-day mortality, and re-admission with AKI, were evaluated. Of the 304 patients included, 69.4% were male. The majority of patients were from the Middle East (65.8%), followed by 28.6% from Asia. Four-hour SLED resulted in a significant improvement in the renal parameters. Recovery from AKI was observed in 25.4%, in-hospital mortality rate was 48.7%, while the 30- and 180-day mortality outcomes were 3.2 and 9.6%, respectively, and re-admission with AKI was observed in 16.9%. Our findings suggest that 4-h SLED significantly improved renal parameters and was associated with favorable clinical outcomes in terms of survival and AKI recovery, suggesting possible utilization of SLED shorter than 6 h in the acute settings to preserve time and manpower for procedures.

## 1 Introduction

Acute kidney injury (AKI) is the rapid deterioration in renal parameters, including serum creatinine (SrCr) and urine output, as defined by the Kidney Disease Improving Global Outcomes (KDIGO) criteria [1]. AKI continued to be a tremendous burden on the healthcare system in terms of mortality, prolongation of hospital stay, and the need to initiate renal replacement therapy (RRT) [2]. It is estimated that 8% of AKI patients require dialysis and the majority usually start the first dialysis session in the intensive care units (ICU) [2]. Furthermore, it is estimated that around 3% of the patients who develop AKI in hospital settings usually end up having long-term dialysis [3].

Hemodialysis was first started early in the 20th century and the spectrum of service has expanded since then worldwide [4,5]. According to previously published data, the most common predisposing factors to AKI are volume depletion and sepsis [6]. Around 7% of these patients ended up with chronic regular dialysis [6]. Over the years, several subtypes of hemodialysis have emerged and been implemented in the clinical practice. There are three main types of dialysis: intermittent hemodialysis (IHD), sustained low-efficiency dialysis (SLED), and continuous RRT (CRRT). Although the indications to start RRT are the same for all modalities, nephrologists may prefer the selection of one modality over another based on individual preferences and the appropriate clinical settings [7].

SLED is characterized by slower dialysate and blood flow rates than IHD [8]. The dialysis sessions in SLED are usually done over 6–12 h and they could be performed on a daily basis [7,9]. This offers better hemodynamic stability for critically ill patients with low mean arterial blood pressure [10]. Compared to CRRT, which is continuous throughout the day, SLED has cost advantages and less exposure to anticoagulation [11]. There are no clear differences among these dialysis modalities in terms of renal recovery or mortality [7].

According to our local practice at Heart Hospital (HH) in Qatar, which is the main tertiary cardiology center in the country, SLED is offered to critically ill patients over a duration of 4 h, rather than the standard 6–12 h. The rationale behind this deviation is to spare time for other procedures needed in the critical care units, better patient’s convenience, efficient utilization of manpower for other services and procedures, and less anticoagulation usage, and hence fewer complications. Nevertheless, to the best of our knowledge, the appropriateness and clinical outcomes of 4-h SLED have not yet been evaluated. Therefore, we conducted a retrospective observational study to explore the renal and clinical outcomes of 4-h SLED among patients admitted to HH over a 4-year period.

## 2 Methods

### 2.1 Study setting

This study was conducted at HH in Qatar. The hospital is the main tertiary cardiology center under Hamad Medical Corporation (HMC), which is the principal public healthcare provider in the country.


**Ethical approval and consent to participate:** The study was approved by HMC Medical Research Centre and the Institutional Review Board (MRC-01-21-036).

### 2.2 Study design and population

We conducted a retrospective observational study involving patients with or without end-stage renal disease (ESRD) admitted to our center and required dialysis which was conducted as 4-h SLED during their hospital stay. The study was approved by the HMC Medical Research Centre and the Institutional Review Board (MRC-01-21-036). The study comprised two stages: (1) determining the change in blood pressure and renal parameters, including SrCr, blood urea nitrogen (BUN), potassium (K^+^), sodium (Na^+^), phosphorus (P), calcium (Ca^++^), and bicarbonate (HCO_3_^−^) before and after 4-h SLED sessions and (2) assessing the clinical outcomes of 4-h SLED, including AKI recovery, in-hospital mortality, 30-day mortality, 180-day mortality, and re-admission with AKI.

### 2.3 Eligibility criteria

Patients were included in the study if they fulfilled the following criteria: (1) adult patients >18 years; (2) hemodynamically unstable ESRD already on IHD prior to admission; (3) hemodynamically unstable AKI or AKI on top of chronic kidney disease (CKD) requiring dialysis; and (4) underwent a minimum of one and a maximum of ten consecutive 4-h SLED session(s) during admission. Patients were excluded if they had one of the following: (1) hemodynamically stable ESRD patients on IHD; (2) did CRRT during the index admission; (3) underwent SLED of less than 4 h or more than 4 h; or (4) underwent isolated ultrafiltration. Eligible patients underwent 4-h SLED which is done at our institution at a blood flow rate of 150 mL/min, dialysate flow of 300 mL/min, and high-flux dialyzer, predominantly without anticoagulation with heparin unless deemed necessary by the treating nephrologist.

### 2.4 Outcome measures and follow-up

The primary outcomes of 4-h SLED were: renal parameters, including SrCr, BUN, K^+^, Na^+^, P, Ca^++^, and HCO_3_^−^ before and after all the 4-h SLED sessions done during the index admission. The secondary outcomes evaluated were: (1) the change in blood pressure before and after all the 4-h SLED sessions; (2) the volume of fluid removed per each SLED session; (3) the need for vasopressor support during SLED; and (4) clinical outcomes including, AKI recovery defined as return of SrCr to baseline value before developing AKI, in-hospital mortality defined all-cause mortality during the index admission, 30-day mortality defined all-cause mortality within 30 days of discharge, 180-day mortality defined all-cause mortality within 180 days of discharge, and re-admission with AKI defined as a rise in SrCr by at least 2 times the baseline value, according to the KDIGO criteria [1]. Patients were followed up for 180 days post-discharge after the index admission.

### 2.5 Data collection procedures

The baseline characteristics of the study participants and the outcomes of interest, including the primary and secondary outcomes as well as patient-, disease-, and medication-related factors, were collected from the HMC electronic medical records system (Cerner^®^) mainly by reviewing the physicians notes documented during the index admission and the results of all laboratory values done during the admission. Moreover, during the 180-day follow-up period, we reviewed the encounters between the patients and other healthcare providers within HMC as all facilities in the corporate have an integrated system. Data collection was conducted from 1 April 2021 to 31 October 2021. Relevant data were manually extracted using a pretested data collection form.

### 2.6 Statistical analyses

Data analyses were performed using the Statistical Package for Social Sciences program version 24.0 (IBM SPSS Statistics for Windows; IBM Corp, Armonk, NY). Descriptive statistics were reported in the form of frequencies and percentages for categorical variables, mean ± standard deviation (SD) for normally distributed continuous variables, and median with interquartile range for skewed continuous variables. The primary analysis was designed to demonstrate the change in the renal parameters, systolic blood pressure (SBP), and diastolic blood pressure (DBP) before and after SLED sessions done during the index admission. The change in all parameters was analyzed using Wilcoxon signed-rank test as all the primary outcome parameters were skewed. A *p*-value of <0.05 was used to indicate statistical significance.

## 3 Results

### 3.1 Subject selection

**Figure 1:**
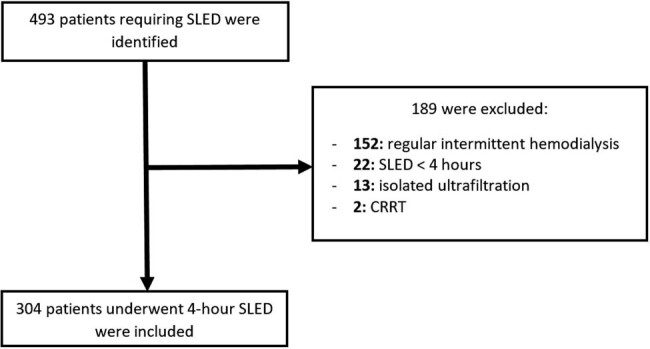
Screening and enrollment of study participants.

We have reviewed 493 patients admitted to HH for different reasons and required at least one session of 4-h SLED throughout their hospital stay. Of these, 189 were excluded for a variety of reasons as demonstrated in Figure 1, and the remaining 304 patients met the eligibility criteria of our study and served as the study cohort.

### 3.2 Baseline characteristics

The baseline characteristics of the subjects (*n* = 304) are presented in Table 1. Male gender represents 69.4% of the subjects. The majority of the subjects were originally from the Middle East and Asia, 65.8 and 28.6% respectively, while only 4.6 and 1% were Africans and Europeans. The most common comorbidities found in the study population were hypertension, diabetes mellitus, and heart failure, 76.3, 74.3, and 70.7%, respectively, followed by CKD and ESRD on regular hemodialysis. Almost 35% were admitted because of acute decompensated heart failure, and about 31% were admitted due to acute coronary syndrome. Patients admitted with acute decompensated heart failure secondary to acute coronary syndrome represented 13.8% of the study population. However, cardiogenic shock was the predominant underlying cause of the hemodynamic instability and it was found in 45.4% of the subjects.

**Table 1:** Baseline characteristics of patients undergoing 4-h SLED (*N* = 304)

Characteristic	*n* (%)
Gender	
Male	211 (69.4)
Female	93 (30.6)
Age (years)	64 [19]
Weight (kg)	74 [22]
Region of origin	
Middle East	200 (65.8)
Asia	87 (28.6)
Africa	14 (4.6)
Europe	3 (1.0)
Medical history	
Hypertension	232 (76.3)
Diabetes mellitus	226 (74.3)
CKD	197 (64.8)
ESRD on regular dialysis	45 (14.8)
Heart failure	215 (70.7)
Reason for hospital admission	
Acute coronary syndrome	94 (30.9)
Acute decompensated heart failure	106 (34.9)
Acute decompensated heart failure due to acute coronary syndrome	42 (13.8)
Elective admission for a cardiac procedure	12 (3.9)
Other reasons	50 (16.5)
Reason for hemodynamic instability	
Cardiogenic shock	138 (45.4)
Complete heart block	2 (0.7)
Mixed septic and cardiogenic shock	31 (10.2)
Septic shock	19 (6.3)
Rhabdomyolysis	2 (0.7)
Other reasons	112 (36.7)
Baseline laboratory values*	
BUN (mmol/L)	13 [16]
SrCr (μmol/L)	183 [180]
Na^+^ (mmol/L)	137 [5]
K^+^ (mmol/L)	4.4 [0.8]
HCO_3_^−^ (mmol/L)	21 [9]
Ca^++^ (mmol/L)	2.2 [0.2]
P (mmol/L)	1.2 [0.3]
Parathyroid hormone	189 [244]
Laboratory values and vitals upon admission*	
BUN (mmol/L)	15 [15]
SrCr (μmol/L)	213 [226]
Na^+^ (mmol/L)	136 [8]
K^+^ (mmol/L)	4.5 [1]
HCO_3_^−^ (mmol/L)	22 [7]
Ca^++^ (mmol/L)	2.3 [0.2]
P (mmol/L)	1.4 [0.7]
Parathyroid hormone (pg/mL)**	420 ± 332
SBP (mmHg)	130 [43]
DBP (mmHg)	71 [20]

*Presented as median [interquartile range].

**Presented as mean ± SD; SLED: sustained low-efficiency dialysis.

The renal parameters, including BUN and SrCr at baseline prior to admission were 13 mmol/L [interquartile range, 16] and 183 μmol/L [interquartile range, 180], respectively. The same parameters were worse upon admission as shown in Table 1.

### 3.3 Effectiveness outcomes

#### 3.3.1 Vitals and renal parameters

**Figure 2:**
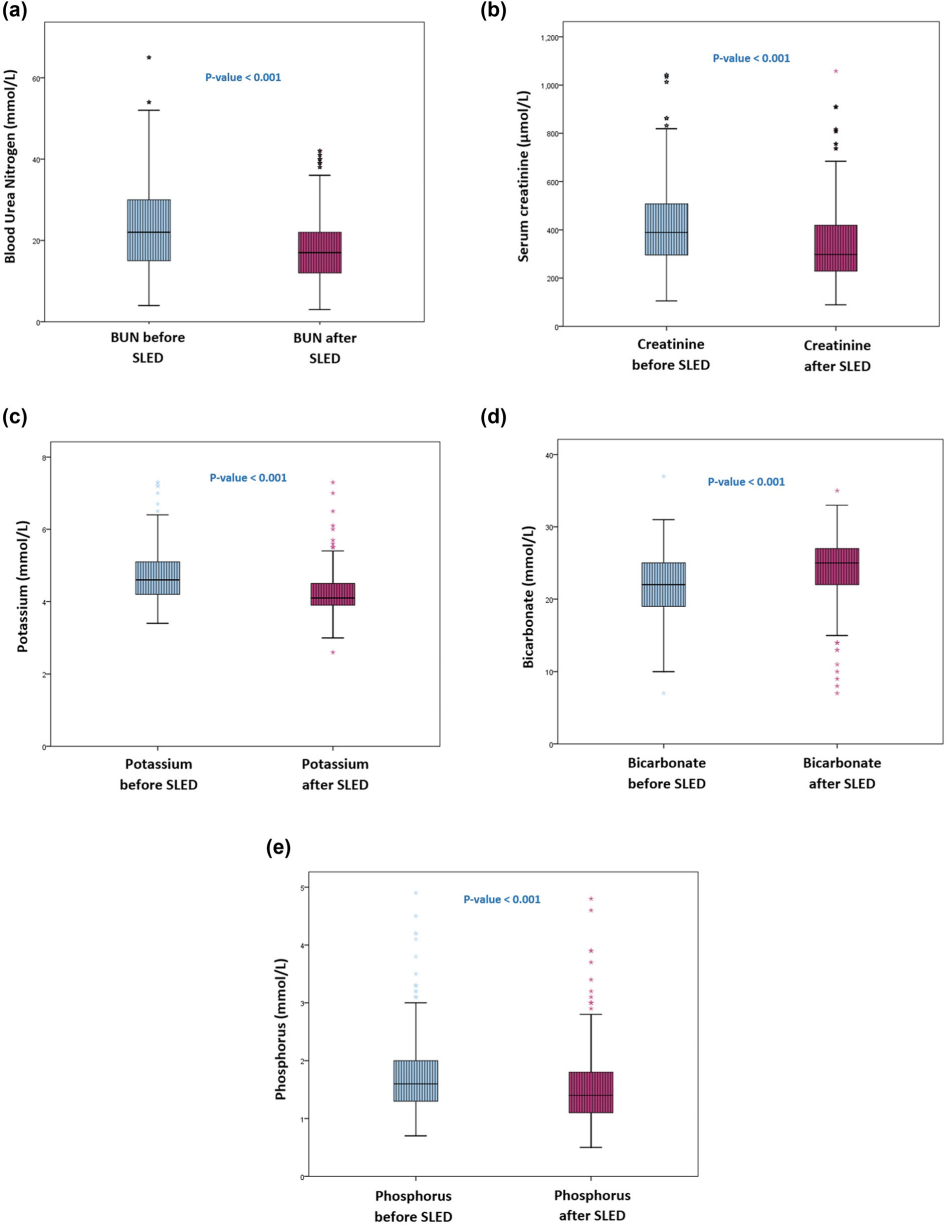
Renal parameters before and after 4-h SLED. (a) Blood urea nitrogen, (b) serum creatinine, (c) potassium, (d) bicarbonate, (e) phosphorus.

The change in blood pressure and the majority of the renal parameters post 4-h SLED sessions was statistically significant as illustrated in Table 2. The BUN decreased from 22 mmol/L [interquartile range, 15] to 17 mmol/L [interquartile range, 10]; *p* < 0.001, and SrCr decreased from 383 μmol/L [interquartile range, 216] to 299 μmol/L [interquartile range, 191]; *p* < 0.001, as demonstrated in Figure 2a and b. Similarly, K^+^, HCO_3_^−^, and P significantly improved with 4-h SLED, as shown in Figure 2c–e. While serum Ca^++^ was the only renal parameter that was not significantly affected by 4-h SLED with a *p*-value of 0.443. The characteristics of the individual SLED sessions are described in Table 3. Vasopressor support was primarily required in the first SLED session. Vasopressors, including noradrenaline, dopamine, and vasopressin were used in 9.9, 4.9, and 1.6% of the patients, respectively. With regard to the anticoagulation use for 4-h SLED, it was rarely used as demonstrated in Table 3. Additionally, SrCr and BUN before and after each session were described, and both parameters improved significantly in the individual SLED sessions except in the tenth SLED session where BUN change did not meet statistical significance.

**Table 2:** Comparison of the renal parameters and vitals before and after 4-h SLED (*N* = 304)

Variable	Pre-SLED*	Post-SLED*	*p* -value
BUN (mmol/L)	22 [15]	17 [10]	*p* < 0.001
SrCr (μmol/L)	383 [216]	299 [191]	*p* < 0.001
Na^+^ (mmol/L)	136 [6]	137 [4]	*p* < 0.001
K^+^ (mmol/L)	4.6 [0.9]	4.1 [0.6]	*p* < 0.001
HCO_3_^−^ (mmol/L)	22 [6]	25 [5]	*p* < 0.001
Ca^++^ (mmol/L)	2.1 [0.2]	2.1 [0.2]	0.443
P (mmol/L)	1.6 [0.8]	1.4 [0.7]	*p* < 0.001
SBP (mmHg)	115 [25]	114 [31]	0.009
DBP (mmHg)	64 [14]	63 [12]	0.001

*Presented as median [interquartile range]; BUN: blood urea nitrogen; SLED: sustained low-efficiency dialysis.

**Table 3:** Characteristics of individual SLED sessions

Parameter	SLED 1 (*n* = 304)	SLED 2 (*n* = 215)	SLED 3 (*n* = 155)	SLED 4 (*n* = 115)	SLED 5 (*n* = 97)	SLED 6 (*n* = 82)	SLED 7 (*n* = 67)	SLED 8 (*n* = 54)	SLED 9 (*n* = 44)	SLED 10 (*n* = 37)
Loop diuretic, *n* (%)										
Furosemide bolus	59 (19.4)	41 (19.1)	23 (14.8)	19 (16.5)	13 (13.4)	8 (9.8)	4 (6.0)	1 (1.9)	4 (9.1)	4 (10.8)
Furosemide infusion	105 (34.5)	37 (17.2)	26 (16.8)	8 (7.0)	4 (4.1)	5 (6.1)	4 (6.0)	3 (5.6)	3 (6.9)	3 (8.1)
Oral furosemide	7 (2.3)	6 (2.8)	3 (1.9)	3 (2.6)	2 (2.1)	3 (3.7)	5 (7.5)	3 (5.6)	2 (4.5)	2 (5.4)
None	133 (43.8)	131 (60.9)	103 (66.5)	85 (73.9)	78 (80.4)	66 (80.5)	54 (80.6)	47 (87.0)	35 (79.5)	28 (75.7)
Loop diuretic TDD (mg)*	235 ± 154	189 ± 158	175 ± 125	157 ± 139	175 ± 139	221 ± 170	168 ± 126	207 ± 151	116 ± 81	120 ± 92
Anticoagulation for dialysis, *n* (%)	4 (1.3)	6 (2.8)	4 (2.6)	2 (1.7)	4 (4.1)	3 (3.7)	3 (4.5)	1 (1.9)	0	0
Vasopressor support during SLED										
Dopamine	15 (4.9)	6 (2.8)	7 (4.5)	1 (0.9)	2 (2.1)	2 (2.4)	0	1 (1.9)	0	2 (5.4)
Noradrenaline	30 (9.9)	6 (2.8)	8 (5.2)	4 (3.5)	5 (5.2)	2 (2.4)	4 (6.0)	4 (7.4)	1 (2.3)	1 (2.7)
Vasopressin	5 (1.6)	3 (1.4)	3 (1.9)	0	1 (1.0)	0	0	0	0	1 (2.7)
Vasopressor dose increased	68 (22.4)	44 (20.5)	24 (15.5)	17 (14.8)	8 (8.2)	8 (9.8)	6 (9.0)	5 (9.3)	4 (9.1)	1 (2.7)
Others	8 (2.6)	8 (3.7)	1 (0.6)	0	1 (1.0)	0	1 (1.5)	0	1 (2.3)	0
None	178 (58.6)	148 (68.8)	112 (72.3)	93 (80.9)	80 (82.5)	70 (85.4)	56 (83.6)	44 (81.5)	38 (86.4)	32 (86.5)
Midodrine, *n* (%)	7 (2.3)	7 (3.3)	9 (5.8)	8 (7.0)	6 (6.2)	10 (12.2)	8 (11.9)	5 (9.3)	5 (11.4)	4 (10.8)
Ultrafiltration volume (L)*	1.45 ± 0.82	1.79 ± 0.90	1.78 ± 0.81	1.96 ± 0.77	2.02 ± 0.66	1.98 ± 0.77	1.96 ± 0.72	2.02 ± 0.80	2.04 ± 0.67	2.04 ± 0.64
SrCr pre-SLED (μmol/L)**	400 [220]	364 [209]	355 [242]	361 [199]	321 [207]	310 [190]	314 [162]	309 [205]	331 [188]	316 [172]
SrCr post-SLED (μmol/L)**	312 [193]	284 [165]	274 [193]	273 [148]	268 [179]	255 [154]	271 [177]	263 [159]	292 [192]	195 [175]
*p* -value	*p* < 0.001	*p* < 0.001	*p* < 0.001	*p* < 0.001	*p* < 0.001	*p* < 0.001	*p* < 0.001	*p* < 0.001	*p* < 0.001	*p* = 0.04
BUN pre-SLED (mmol/L)**	26.3 [20]	17.7 [18]	23.4 [14]	20.2 [14]	19.0 [12]	17.1 [13]	16.1 [12]	17.1 [11]	16.3 [11]	15.8 [13]
BUN post-SLED (mmol/L)**	18.6 [13]	15.8 [11]	16.8 [11]	10.8 [3]	15.7 [11]	14.1 [9]	15.1 [10]	13.8 [9]	14.7 [9]	15.7 [10]
*p* -value	*p* < 0.001	*p* < 0.001	*p* < 0.001	*p* < 0.001	*p* < 0.001	*p* < 0.001	*p* < 0.001	*p* < 0.001	*p* = 0.007	*p* = 0.159

*Presented as mean ± SD.

**Presented as median [interquartile range]; SLED: sustained low-efficiency dialysis; TDD: total daily dose; BUN: blood urea nitrogen.

#### 3.3.2 Clinical outcomes

Around half of the study population (*n* = 130, 48.1%) were successfully discharged from the ICU as shown in Table 4. In terms of AKI recovery, around a quarter of the study population (*n* = 66, 25.4%) achieved this outcome, while 25 (16.9%) were re-admitted with AKI. On the other hand, the in-hospital mortality was observed in 48.7% of the study population, while the 30- and 180-day mortality outcomes were 3.2 and 9.6% among those who were successfully discharged from hospital, respectively.

**Table 4:** Outcomes of 4-h SLED (*N* = 304)

Outcome	*n* (%)
AKI recovery*	66 (25.4)
Discharge from ICU**	130 (48.1)
In-hospital mortality	148 (48.7)
30-Day mortality^§^	5 (3.2)
180-Day mortality^§^	15 (9.6)
Re-admission with AKI^‡^	25 (16.9)

*Excluding 45 patients who had ESDR at baseline.

**Excluding 34 patients who didn’t require ICU admission. ^§^Excluding patients who died during hospital stay. ^‡^Excluding patients who died either during the hospital stay or within 6 months of discharge; SLED: sustained low-efficiency dialysis; AKI: acute kidney injury; ICU: intensive care unit.

## 4 Discussion

The benchmark is to perform SLED over 6–12 h among hemodynamically unstable patients who require dialysis [7,9]. Nevertheless, at our center, SLED is performed over 4 h, which is considered shorter than the ideal SLED duration for a variety of reasons, including sparing time for other procedures needed in the critical care units, making the procedure more convenient to the patient and healthcare providers, utilizing the manpower for other services and procedures, and reducing the exposure to anticoagulation during dialysis. Therefore, we opted to evaluate the effectiveness of 4-h SLED. Our retrospective analysis over 4 years demonstrated that performing 4-h SLED among patients with hemodynamic instability requiring dialysis significantly improved the renal parameters, including SrCr, BUN, K^+^, and HCO_3_^−^, and was associated with favorable short-term and long-term outcomes, including 30-day and 180-day mortality post-discharge.

We found that SLED as short as 4 h improved SrCr, BUN, Na^+^, K^+^, HCO_3_^−^, and P among hemodynamically unstable patients requiring dialysis. Our findings were similar to the findings reported by Marshall et al. who demonstrated that 6–12-h SLED with a mean of 10.4 h, resulted in a significant reduction in SrCr, BUN, K^+^, and HCO_3_^−^ [12]. Likewise, a retrospective study of 91 patients with AKI treated with SLED that was conducted over 8–12 h showed that SLED significantly improved BUN, SrCr, and K^+^ within 24 h of SLED [13]. Other studies had evaluated the appropriateness and adequacy of SLED in comparison to other dialysis modalities by assessing Kt/V and urea reduction ratio (URR); however, we have not calculated Kt/V or URR as these calculations would require BUN to be withdrawn directly post-dialysis, which is not routinely done in our daily practice, and these calculations are of less utility for the evaluation of RRT adequacy in the acute settings, which resemble our study settings [14,15,16,17].

There is still a lack of solid evidence favoring a mode of RRT over another in patients with AKI in terms of survival [7]. We have demonstrated that the use of 4-h SLED resulted in an in-hospital mortality of 48.7%. Our observed mortality rate was lower than the rate reported by Marshall and colleagues in 2001 who showed that the in-hospital mortality of SLED conducted over a mean of 10.4 h was 62.2% [12]. Nevertheless, 3 years later, Marshall et al. reported that the observed in-hospital mortality of 8-h SLED was 46% among 24 critically ill patients, which is similar to the mortality rate we observed in our retrospective analysis [15]. AKI recovery was observed in 25.4% of our study population. In a retrospective observational study that compared 8-h SLED and CRRT in 232 patients with AKI, short-term RRT dependence was comparable between SLED and CRRT [11]. Additionally, we found that the 30-day mortality post-discharge among patients receiving 4-h SLED was 3.2% only. In comparison to other literature evaluating SLED clinical outcomes, our reported 30-day mortality is considered low. Kitchlu et al. reported that the 30-day mortality was 54% in 74 patients treated with 8-h SLED [11]. Similarly, a retrospective study of 91 patients with AKI treated with 8–12 h SLED showed that mortality at 1 month was 58% [13]. This huge variation in the 30-day mortality rate between our study and previous literature could be explained by separating the outcomes into in-hospital mortality and 30-day mortality in our study, while the two studies previously discussed combined the two outcomes; thus, their 30-day mortality was inclusive of in-hospital mortality as well [11,13]. On top of 30-day mortality, we have evaluated the long-term effect of 4-h SLED by assessing the 180-day mortality and we found it to be 9.6%. To the best of our knowledge, this is the first study to explore 180-day mortality as a long-term outcome of SLED.

This study was an observational retrospective study that has its inherent limitations. First, the retrospective nature of the study with the dependence on the electronic medical records to obtain the study data carries a risk of missing important information that may not be appropriately documented in the medical records. Second, to assess the adequacy of 4-h SLED, we have relied solely on the change in renal parameters withdrawn on the day of dialysis before SLED and within 24 h after SLED without calculating Kt/V or URR. The calculation of Kt/V or URR was not feasible, especially that in our institution, BUN is not withdrawn directly after dialysis, and our study was conducted in acute settings where Kt/V and URR are of less utility. Third, our study did not compare 4-h SLED to the standard SLED of 6–12 h due to underutilization of standard SLED at our institution. Nevertheless, this retrospective observational study, despite the previous limitations, had a good number of participants and most importantly, tried to answer a unique question and fill a gap in the existing literature to determine whether performing SLED with a shorter duration than the standard duration would achieve favorable renal and clinical outcomes.

## 5 Conclusion

This study suggests that the use of 4-h SLED significantly improved renal parameters among patients with hemodynamic instability requiring dialysis, which might encourage utilizing SLED shorter than the standard duration to preserve time and manpower for essential procedures during the hospital stay. Prospective trials comparing 4-h SLED with standard SLED need to be conducted to confirm the findings of our study.
